# The health-related quality of life in rheumatoid arthritis, ankylosing spondylitis, and psoriatic arthritis: a comparison with a selected sample of healthy people

**DOI:** 10.1186/1477-7525-7-25

**Published:** 2009-03-18

**Authors:** Fausto Salaffi, Marina Carotti, Stefania Gasparini, Michele Intorcia, Walter Grassi

**Affiliations:** 1 Dipartimento di Patologia Molecolare e Terapie Innovative, Clinica Reumatologica – Università Politecnica delle Marche, Ancona, Italy; 2 Dipartimento di Radiologia, S.O.D. Radiologia Clinica – Università Politecnica delle Marche, Ancona, Italy; 3 Global Epidemiology and Outcomes Research, Bristol-Myers Squibb, Roma, Italy

## Abstract

**Background:**

The health-related quality of life (HRQL) is an important indicator of the burden of musculoskeletal disease. The Medical Outcome Study Short-Term 36 (SF-36) is the most used tool that evaluates HRQL as a subjective perception about psychological and physical limitations due to an underlying illness. The purpose of this study was to compare the HRQL scores among patients with rheumatoid arthritis (RA), psoriatic arthritis (PsA) and ankylosing spondylitis (AS) and a selected sample of health people and determine their relationship with measures of clinical condition.

**Methods:**

799 patients (469 with RA, 164 with AS, 65 with axial PsA and 101 with peripheral PsA) accepted the invitation to participate. 1579 healthy controls were used for the comparison. We calculated scores for the eight SF-36 subscales, the Physical Component Summary (PCS) score, and the Mental Component Summary (MCS) score, according to published algorithms. Disease-related characteristics included disease duration, comorbidity, a measure for disease activity and for radiographic damage. The presence of comorbidity was ascertained through patient's self-reports by the Self-Administered Comorbidity Questionnaire (SCQ). Comparison were performed with respect to sex and age, and s-scores were calculated for comparison with the norm. Multivariate analyses were used to assess the relationship between HRQL and radiographic damage, disease activity, and socio-demographic data.

**Results:**

The four inflammatory rheumatic diseases (IRD), compared to controls, significantly impaired all eight health concepts of the SF-36 (p < 0.0001) in both component PCS and MCS scores (p < 0.0001). Overall, the dimensions typically affected were physical functioning, limitations due to physical function, and bodily pain. The disease with the worst HRQL for those dimensions was RA. The multivariate analyses revealed that the physical component was influenced by a high disease activity and comorbidity. The severity of psoriatic lesions was associated with poor mental functioning in patients with PsA.

**Conclusion:**

Chronic IRD have a clearly detrimental effect on the HRQL in both sex and in age groups, and physical domain is more impaired than mental and social ones.

## Background

Rheumatoid arthritis (RA), ankylosing spondylitis (AS), and psoriatic arthritis (PsA) are three common types of inflammatory rheumatic diseases (IRD) associated with deformities and joint destruction. RA is the most frequent IRD, with a prevalence of 0.5% [1]. Patients with active RA have been shown to suffer deficits in health-related quality of life (HRQL) along a number of physical functioning and mental health dimensions [2,3]. Furthermore, patients with RA who have significant functional disability have a 3-fold increased risk of mortality compared with that of the general population [4]; this risk is comparable with that of individuals of the general population in the highest quintile for systolic and diastolic blood pressure, cholesterol level, or pack-years of smoking [5]. AS is a systemic and IRD predominantly affecting the axial skeleton with sacroiliac joint involvement as its hallmark, causing decreased spinal mobility [6]. Similarly to other chronic diseases, AS can affect quality of life, morbidity, mortality, participation in paid and unpaid work, and healthcare costs [7-9]. PsA is an inflammatory peripheral and/or axial arthritis associated with psoriasis, usually seronegative for rheumatoid factor [10]. In addition to the peripheral joint disease, patients with PsA have a debilitating skin disease, and up to 50% may also have spinal disease [11]. Compared to RA and AS, there is less information about the burden of illness in PsA. Although considered a benign disease in the majority of cases given in previous reports or in population-based samples [12]. clinical cohort studies described PsA as a progressive, disabling disease, particularly when polyarticular peripheral arthritis is present [10,11,13]. Thus, IRD represents a tremendous economic burden, not only for patients and their families, but also for society as a whole.

Traditional methods of evaluation, with a focus on the locomotor system and measures of impairment, may fail to describe the extensive multi-dimensional issues associated with chronic rheumatic conditions. Consideration of HRQL has become increasingly important in decisions regarding resource allocation, intervention design, and pharmacological treatment with biologic agents of individuals with chronic inflammatory disabling conditions [14-16]. Two broad approaches to measuring patient perceptions of HRQL can be described: generic instruments that provide a broad summary of HRQL, and specific instruments that focus on issues of relevance to a specific disease or patient group. Generic instruments are not age-, disease- or treatment specific, and contain multiple HRQL concepts of relevance to patients and the general population, supporting application in both populations [17]. The Short Form 36-item Health Survey Questionnaire (SF-36) is a widely used example of a generic health profile [18]. The items cover eight domains of HRQL, including physical and social functioning and mental health.

The main objective of this study was to examine the self-reported health status in patients with RA, AS and PsA, compared with a selected sample of health people. Furthermore, we wanted to explore the associations between health status and age, sex of the patients, and educational level in these IRD and to estimate the burden of the disease by controlling for the normal variations in health status in the general population.

## Methods

### Patients

Participants at this study are part of an ongoing longitudinal project measuring rheumatic disease outcomes, approved by the local Ethical Committee for Medical Research. Consecutive adult rheumatic disease patients from the Rheumatology Clinic of the Università Politecnica delle Marche, who agreed to participate in the study, completed an informed consent form. The study population includes patients examined by two rheumatologists and fulfilling the American College of Rheumatology (ACR) classification criteria for RA [19], the modified New York criteria for AS [20], and the European Spondylarthropathy Study Group (ESSG) preliminary criteria for PsA [21]. For the purposes of the present study, AS patients with peripheral articular involvement were excluded. Peripheral involvement was defined as synovitis of at least one large joint (wrist, elbow, shoulder, hip, knee, ankle) or three or more small joints (hands, feet, sternoclavicular joints) [7]. The diagnoses of PsA are recorded with a thesaurus specific for the database. Two terms for PsA have been used: "predominantly peripheral arthritis with psoriasis" (in this report: peripheral PsA) and "predominantly spondarthritis with psoriasis" (axial PsA) [22]. The distinction was made by each treating rheumatologist according to their clinical judgment. Patients with rheumatoid factor positivity and with symmetrical polyarthritis who satisfied the ACR classification criteria for RA were excluded. Information on the presence of psoriasis in familial subjects was also obtained, especially in patients who had features of spondyloarthritis, such as enthesitis. Of the 1121 patients with IRD invited to undergo a complete medical history, a careful clinical examination and radiological evaluation, 799 (71.3%) patients (469 with RA, 164 with SA, 65 with axial PsA and 101 with peripheral PsA) accepted the invitation to participate by completing the questionnaires and the physical and radiological evaluation. For comparison, data from a previous cross-sectional population-based study, namely MAPPING (MArche Pain Prevalence INvestigation Group) Study will be used. This study design has been described in detail elsewhere [1]. The sample reflects the age/sex related stratification/distribution of the Italian population. Briefly, the MAPPING study was conducted on 4000 subjects aged 18 years and over, selected from the practice lists of 16 general practitioner-GPs (total target adult population of 20882 individuals). These GPs were representative of the practices in Marche in terms of size of practice, geographical location, and socio-economic status of those attending. The sample for the survey was selected randomly so that there would be equal numbers from each of the age-sex bands (five age-groups ranging from 18–34 years to 75 years and over) and was weighted to ensure an equal representation of patients in each of the subgroups. A total of 336 individuals were excluded through this procedure: 43 individuals had left the practice, 49 had dementia or mental illness, 31 were terminally ill, 114 had died, and 99 individuals had no reason given. The remaining 3664 individuals were sent a standardized self-completion postal questionnaire. Subjects who did not return their questionnaires within three weeks were sent another questionnaire to maximise the response rate. The patients were instructed to complete all the questionnaires at home and to return them in a prepaid envelope. To increase the response rate the nonresponders were contacted by telephone and encouraged to return the questionnaires. Of 3470 questionnaires delivered (194 participants could not be contacted because of unknown address or recent death, absent from the community during the survey, hospitalization etc.), 2155 were returned after two postal reminders, which gave a response rate of 62.1%. Of these 2155 people who completed the questionnaires, 576 subjects were diagnosed as having had rheumatic disease at the time of the study [1]. The data collected from the remaining 1579 health controls were used in this study.

### Demographics, disease-related characteristics, quality of life assessment, and radiographic scoring methods

A comprehensive questionnaire package including socio-demographic data, quality of life items, and disease-related variables was administered to the patients. The socio-demographic variables were age, sex, and highest attained level of education (primary; secondary; high school/university). Disease-related characteristics included disease duration (years since fulfilment of the classification criteria of the IRD), comorbidity, a measure for disease activity and for radiographic damage. The Disease Activity Score (DAS) [23] was used to evaluate disease activity in patients with RA and peripheral PsA and the Bath Ankylosing Spondylitis Disease Activity index (BASDAI) [24] was used for patients with AS and axial PsA. DAS has been developed to provide a measure of RA disease activity that is more informative than the several disease activity variables individually [23]. The DAS combines information from the Ritchie articular index; a 44-joint swollen joint count, erythrocyte sedimentation rate, and a general health assessment on a visual analog scale (VAS) [23]. Disease activity in patients with AS and axial PsA was measured with the BASDAI [24]. The BASDAI consists of 6 VAS relating to major symptoms relevant to AS: fatigue, spinal pain, joint pain, localized tenderness, and morning stiffness (measured in terms of both degree and length of time stiffness persists). The BASDAI items range from none (0) to very severe (100) symptoms [24]. The mean score of 5 items (mean of the 2 morning stiffness items plus the 4 remaining items) is applied as an estimate of disease activity. Information about HRQL was obtained with a validated Italian translation of the self-administered SF-36 (IQOLA SF-36 Italian Version 1.6) [25]. The SF-36 contains 36 items, organized into eight scales covering the dimensions physical functioning (PF), role limitations due to physical function (RP), bodily pain (BP), general health (GH), mental health (MH), role limitations due to emotional health (RE), social functioning (SF), and vitality (VT). One additional item pertains to health transition [18]. The raw scores were coded and recalibrated following the standard guidelines, and the items were then summed and transformed to the eight 0–100 scales (0 = worst health, 100 = best health) [18]. On the basis of these separate subscales, component summary scores can be calculated to provide a global measure of physical (PCS) and mental functioning (MCS) [26]. The PCS and MCS scores range from 0–100, with higher scores indicating better health [26]. Radiographic damage was assessed, by a single radiologist (MC) who was unaware of patient identity, using three different scoring methods. Radiographs of the hands and feet were assessed in RA patients, using the modified Sharp/van der Heijde method [27]. Inter-observer agreement was tested by a second investigator (FS) on 20 sets of radiographs and the intra-class correlation coefficient between the two investigators was 0.91. The Sharp van der Heijde modified scoring method [28] was used for assessing erosions and joint space narrowing of joints of hands and feet in peripheral PsA. The proposed adapted scoring method for PsA is a detailed scoring method evaluating erosions, joint space narrowing, (sub)luxation, ankylosis, gross osteolysis, and pencil in cup phenomena. The modified Stoke Ankylosing Spondylitis Spine Score (mSASSS) [29] scoring system was used to analyse the conventional *x*-ray findings in patients with AS and with axial PsA. The mSASSS offers advantages in measurement properties and is the most appropriate method by which assessing progression of structural damage in AS [30]. The severity of psoriatic lesions was also assessed, using the Psoriasis Area and Severity Index (PASI) [31]. The PASI is a composite score used to evaluate the severity of psoriatic lesions by assessing the extent of skin involvement, erythema, plaque thickness, and degree of scaling [31]. The PASI score can vary in increments of 0.1 units from 0 to 72, with higher scores representing a greater degree of psoriatic severity. Finally, the presence of comorbidity was ascertained through patient's self-reports using the Self-Administered Comorbidity Questionnaire (SCQ) [32], an efficient method to assess comorbid conditions in clinical and health services research. The SCQ is short, easily understood, and can be completed by individuals without any medical background. It also allows the subject to note the severity of each comorbid condition and their perception of its impact on their function. Because there are 12 defined medical problems and 3 optional conditions (1 point for the presence of the problem, another point if he/she receives treatment for it, and an additional point if the problem causes a limitation in functioning) the maximum score totals 36 points are used [32].

### Statistical analysis

The data were analysed using the SPSS version 11.0 (SPSS Inc, Chicago, IL), and MedCalc^®^, version 9.2 for Windows XP. Descriptive statistics are given as means and standard deviations (SD) for continuous data or as percentages for counts. Comparisons between groups were performed with chi-square tests for categorial variables and analysis of variance (ANOVA) for continuous variables. Standardized difference scores (the s-score or normal score) were also calculated by subtracting the mean scores of the patients from the mean scores of the general population, followed by the division of these deviations by each scale's standard deviation in the general population. The standardized s-score is a rescaled score with a population average of 0 and a standard deviation of 1. The values of the s-scores were interpreted according to Cohen's effect size index, in which 0.2 refers to a small difference, 0.5 to a moderate difference, and 0.8 or more to a large difference [33]. A set of multivariable analyses were constructed to adjust for factors potentially associated with poor HRQL in the four IRD groups. Covariates chosen *a priori *included sex (as a dichotomous variable; 0 = male; 1 = female); age (as a continuous variable); disease duration (years from disease onset as a continuous variable); educational level (years of education as a continuous variable); and the average score of the SCQ questionnaire (SCQ score as a continuous variable). All these factors were then introduced as covariates in multiple regression models in which PCS and MCS SF-36 scores were dependent variables. All variables were entered simultaneously. Owing to multiple comparisons with increasing risk of type 1 errors, the level of statistical significance was set at 0.01.

## Results

### Demographic and clinical data

Demographics and disease characteristics of patients and healthy controls enrolled in the study are shown in Table 1. There was no significant difference in the demographics and disease characteristics of those completing (799 patients) and refusing to complete the questionnaires and the clinical and radiological evaluation (322 patients), and no significant difference in the proportions of men and women. As expected, our RA patients were older and predominantly female, whereas AS patients were younger and predominantly male, respect to the general population. The age and sex distributions of the patients with RA and PsA and those with AS are significantly different (p < 0.001). Slightly more than one quarter of the patients with RA, more than two thirds of the patients with AS, and slightly less than an half of the patients with PsA (44.6 with peripheral PsA and 49.3 with axial PsA) were male. The onset of AS is typically earlier than RA; therefore, in older age-matched healthy controls, the patients with AS will have a longer disease duration than those with RA or PsA. The educational level among patients with RA was lower than among patients with AS and PsA (p < 0.01). Approximately, more than an half of our chronic IRD patients reported some comorbidity (hypertension, heart diseases, gastrointestinal conditions, and chronic respiratory diseases were the 4 most prevalent comorbidities). Statistically significant differences were found for a number of comorbid conditions (p < 0.001) and average score of the SCQ questionnaire (p < 0.001). Compared with the general population, significantly higher prevalence estimates were observed with respect to cardiovascular disorders (p < 0.001), chronic pulmonary disease (p < 0.01), and gastrointestinal diseases (p < 0.001).

**Table 1:** Characteristics of patients with rheumatoid arthritis (RA), ankylosing spondylitis (AS), psoriatic arthritis (PsA) and the general population (healthy controls)

	Rheumatoid arthritis (n = 469)	Ankylosing spondylitis (n = 164)	Peripheral psoriatic arthritis (n = 101)	Axial psoriatic arthritis (n = 65)	General population (n = 1579)
**Women (%)**	71.8	18.9	61.4	50.7	50.2
					
**Age (years)**					
**- mean (± SD)**	57.5 (14.3)	51.7 (9.2)	60.7 (11.6)	58.2 (10.3)	55.2 (19.2)
					
**Disease duration**					
**- mean (± SD)**	6.1 (4.2)	8.2 (4.6)	7.5 (5.3)	8.4 (4.3)	NA
					
**Educational level, n (%)**					
**- primary school**	240 (51.2)	70 (42.7)	45 (44.6)	30 (46.1)	928 (58.8)
**- secondary school**	149 (31.8)	65 (39.6)	43 (42.6)	25 (38.5)	418 (26.5)
**- high school/university**	80 (17.0)	29 (17.7)	13 (12.8)	10 (14.4)	233 (14.7)
					
**No of comorbid conditions, n (%)**					
**- none**	217 (46.3)	73 (44.5)	45 (44.6)	19 (29.3)	548 (34.7)
**- 1**	131 (27.9)	37 (22.6)	26 (25.7)	12 (18.5)	334 (33.5)
**- 2**	47 (10.0)	30 (18.3)	13 (12.9)	21 (32.3)	112 (7.1)
**- 3**	20 (4.3)	15 (9.1)	15 (14.8)	10 (15.4)	69 (4.4)
**- 4**	25 (5.3)	6 (3.7)	2 (2.0)	2 (3.0)	21 (1.3)
**- 5 or more**	29 (6.2)	3 (1.8)	0 (0)	1 (1.5)	12 (0.8)
					
**Comorbidity score by SCQ**	4.35 (3.1)	2.48 (1.9)	3.75 (2.5)	3.34 (2.1)	1.95 (1.9)
**- mean (± SD)**					
					
**DAS**					
**- mean (± SD)**	4.5 (0.8)	--	4.4 (0.9)	--	NA
					
**BASDAI**	--	54.7 (17.2)	--	53.8 (14.4)	NA
**- mean (± SD)**					
					
**Rx Sharp score**	59.4 (32.9)	--	62.1 (39.1)	--	NA
**- mean (± SD)**					
					
**mSASSS**					
**- mean (± SD)**	--	14.7 (5.2)	--	13.7 (5.1)	NA
					
**PASI**					
**- mean (± SD)**	--	--	7.1 (3.3)	6.7 (2.9)	NA

NA = non applicable

Abbreviations: DAS = Disease Activity Score; BASDAI = Bath Ankylosing Spondylitis Disease Activity index; mSASSS = modified Stoke Ankylosing Spondylitis Spine Score; PASI = Psoriasis Area and Severity Index

### Self-reported health status results

**Figure 1:**
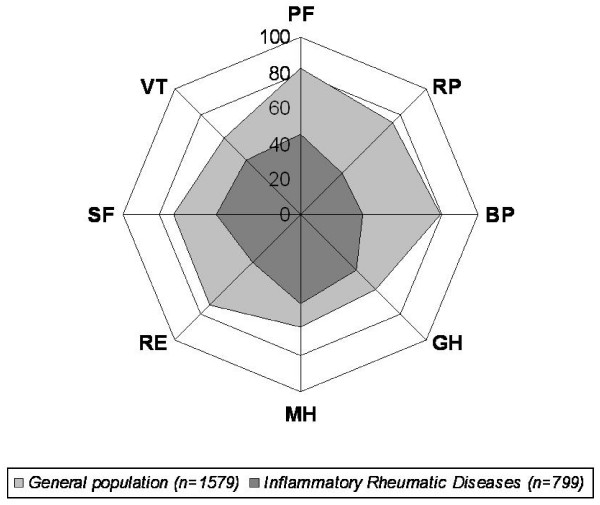
**Comparison of Medical Outcomes Short Form-36 health survey domain scores between patients with inflammatory rheumatic diseases (IRD) and general population normative data**. Higher scores represent better health status. Physical functioning (PF), Role function – Physical aspect (RP), Bodily Pain (BP), General health perception (GH), Mental Health (MH), Role function – Emotional aspect (RE), Social functioning (SF), and Vitality (VT).

**Figure 2:**
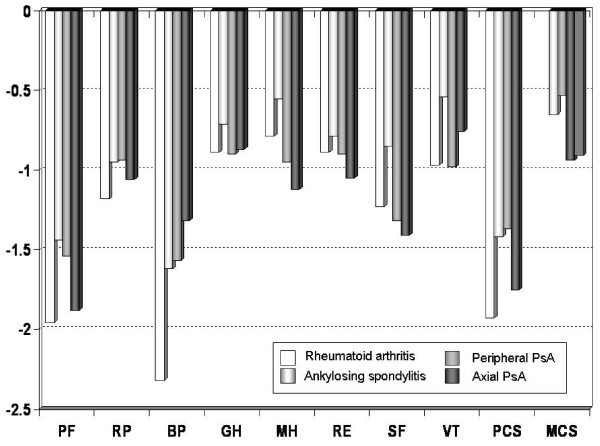
**Standard difference scores (s-scores) for patients with rheumatoid arthritis, ankylosing spondylitis, peripheral PsA and axial Psa**. The values of the s-scores were interpreted according to Cohen's effect size index, in which 0.2 refers to a small difference, 0.5 to a moderate difference, and 0.8 or more to a large difference. Physical functioning (PF), Role function – Physical aspect (RP), Bodily Pain (BP), General health perception (GH), Mental Health (MH), Role function – Emotional aspect (RE), Social functioning (SF), and Vitality (VT), component summary scores of physical (PCS) and mental functioning (MCS).

Scores for respondents with IRD significantly impaired all eight health concepts of the SF-36 (p < 0.0001) and in both component summary scores (PCS and MCS) (p < 0.0001), compared with their non-arthritic counterparts (Table 2). Generally, respondents with IRD report relatively greater deficits in the scales that primarily measure functional disability, i.e., physical functioning, role limitations due to physical function, bodily pain, general health, rather than the scales measuring a construct of mental health, i.e., mental health, role limitations due to emotional health, social functioning, and vitality. The SF-36 scores decreased (indicating a linear decline in HRQL), especially in the physical dimension, with increasing age in all categories of IRD (Table 3). However, HRQL is affected by even in the general population (Table 3). Significant differences were found between men and women only in AS group concerning role limitations due to physical function (p = 0.011), and for general health (p = 0.031), with women reporting worse health than men. No differences were found for the remaining scales. Additionally, patients and controls with high education level reported better health on all subscales of the SF-36 than less educated groups (Table 4). Figure 1 compares the scores in each domain of the SF-36 health survey for the study population to age-adjusted general population norms. The scores for every domain of the SF-36 health survey were lower for the study population than the corresponding age-adjusted norms (Table 2). The quality of life patterns for the different IRD, expressed as standardized s-scores (the difference in the number of standard deviations from the population mean), are shown in Figure 2. Overall, the dimensions typically affected by IRD were physical functioning, limitations due to physical function, and bodily pain. The disease with the worst HRQL for those dimensions was RA. The mean PCS score of RA patients was 32.5 (SD = 5.9). The mean MCS score of patients was 39.4 (SD = 11.8). Regarding the HRQL dimensions involving mental health problems, patients with PsA (both peripheral and axial PsA) score generally lower than the health controls. In patients with AS the physical domain due to role function-physical aspect and bodily pain is more impaired than the mental one.

**Table 2:** Mean ± SD (standard deviation) and 95% CI (confidence intervals) SF-36 scores in patients and the general population*

	**Groups**									
	**Rheumatoid arthritis** (n = 469)		**Ankylosing spondilitis** (n = 164)		**Peripheral PsA** (n = 101)		**Axial PsA** (n = 65)		**General population** (n = 1579)	
	*Mean ± SD*	*95% CI*	*Mean ± SD*	*95% CI*	*Mean ± SD*	*95% CI*	*Mean ± SD*	*95% CI*	*Mean ± SD*	*95% CI*

**PF**	41.8 ± 20.6	39.9–43.6	52.6 ± 21.2	49.4–55.9	43.5 ± 21.4	39.3–47.7	50.6 ± 18.6	46.0–55.2	82.5 ± 20.0	81.9–83.9
**RP**	29.8 ± 16.0	28.3–31.2	38.2 ± 29.7	33.6–42.8	34.3 ± 27.3	28.9–39.7	38.4 ± 26.8	31.8–45.1	73.1 ± 36.7	71.3–74.9
**BP**	30.1 ± 17.0	28.5–31.6	45.0 ± 17.4	42.3–47.7	36.3 ± 17.9	32.7–39.8	45.9 ± 16.9	41.8–50.1	78.5 ± 20.8	77.5–79.6
**GH**	44.0 ± 19.7	42.3–45.8	47.2 ± 22.6	43.7–50.7	45.1 ± 16.8	41.8–48.5	43.8 ± 16.4	39.8–47.9	60.1 ± 18.1	59.3–61.0
**MH**	50.3 ± 23.3	48.2–52.4	54.3 ± 20.8	51.1–57.5	44.7 ± 18.0	41.2–48.3	47.6 ± 20.6	42.5–52.7	63.6 ± 16.8	62.9–64.5
**RE**	38.2 ± 41.4	34.4–41.9	42.0 ± 27.5	37.7–46.2	28.0 ± 29.7	22.1–33.9	37.6 ± 27.4	30.8–44.4	72.1 ± 38.1	70.2–73.9
**SF**	46.9 ± 21.3	45.0–48.8	54.7 ± 20.9	51.5–58.0	43.1 ± 19.2	39.3–46.9	44.7 ± 11.9	41.7–47.7	71.6 ± 20.1	70.6–72.7
**VT**	41.9 ± 20.8	40.1–43.8	48.5 ± 18.6	45.6–51.4	45.1 ± 15.8	42.0–48.3	41.8 ± 19.2	37.0–46.5	56.8 ± 15.4	56.2–57.7
**SF-36 PCS**	32.5 ± 6.0	31.9–33.0	37.1 ± 8.6	35.7–38.4	34.1 ± 6.9	32.8–35.5	37.5 ± 7.0	35.8–39.2	49.6 ± 8.9	49.2–50.2
**SF-36 MCS**	39.4 ± 11.8	38.3–40.5	40.7 ± 9.5	39.2–42.1	36.9 ± 6.8	35.5–38.3	36.5 ± 8.0	34.5–38.5	45.6 ± 8.4	43.1–46.1

*All differences between patients and the general population were significant at p < 0.0001.

Abbreviations: Physical functioning (PF), Role function – Physical aspect (RP), Bodily Pain (BP), General health perception (GH), Mental Health (MH), Role function – Emotional aspect (RE), Social functioning (SF), Vitality (VT), component summary scores of physical (PCS) and mental functioning (MCS)

**Table 3:** SF-36 subscales and summary scores in patients and the controls by age groups. Data are expressed as means ± SD and 95% CI

	***AGE Group (years)***									
	**18 – 34 years**		**35 – 44 years**		**45 – 64 years**		**65 – 74 years**		**> 75 years**	
	*Mean ± SD*	*95% CI*	*Mean ± SD*	*95% CI*	*Mean ± SD*	*95% CI*	*Mean ± SD*	*95% CI*	*Mean ± SD*	*95% CI*

***PF***										
Controls	94.7 ± 10.9	93.4–96.0	91.8 ± 12.1	90.2–93.4	86.6 ± 15.6	85.3–88.0	74.8 ± 19.2	72.5–77.2	65.0 ± 22.7	62.5–67.5
RA	40.0 ± 17.1	34.2–42.7	41.0 ± 18.9	36.2–45.8	41.9 ± 20.7	39.9–45.0	40.6 ± 21.4	36.9–44.3	38.7 ± 21.2	33.2–44.1
SA	47.0 ± 20.3	28.2–65.7	53.5 ± 18.3	47.0–60.0	54.6 ± 20.5	50.5–58.6	49.7 ± 27.7	35.5–63.9	30.0 ± 17.6	11.5–48.5
Per PsA	75.0 ± 10.8	57.8–92.2	59.0 ± 23.1	42.5–75.5	41.5 ± 20.5	34.8–48.1	41.0 ± 20.8	33.7–48.2	35.3 ± 13.9	27.3–43.4
Ax PsA	72.5 ± 16.4	62.2–82.8	58.9 ± 19.5	44.0–73.9	48.3 ± 19.8	40.7–55.8	45.0 ± 14.7	38.3–51.7	62.5 ± 23.5	30.7–94.3
***RP***										
Controls	88.6 ± 24.9	85.6–91.5	85.6 ± 24.5	82.4–88.8	78.6 ± 33.3	75.7–81.6	61.3 ± 38.4	56.6–66.1	51.8 ± 40.4	47.4–56.3
RA	29.3 ± 11.7	22.4–30.2	28.2 ± 15.3	24.3–32.1	29.5 ± 16.0	28.2–32.9	29.7 ± 16.9	27.8–3.7	28.0 ± 17.1	24.6–33.5
SA	46.4 ± 36.6	12.5–80.2	47.1 ± 24.8	38.3–55.9	35.3 ± 28.9	29.5–41.0	44.1 ± 38.0	24.5–63.6	32.5 ± 19.6	30.8–36.8
Per PsA	56.2 ± 31.4	16.1–86.3	47.5 ± 27.5	27.8–67.1	30.5 ± 28.1	21.4–39.6	29.0 ± 26.3	19.8–38.2	41.6 ± 20.6	29.7–53.5
Ax PsA	50.0 ± 20.4	17.5–82.4	50.0 ± 35.3	22.8–77.1	33.6 ± 26.1	23.6–43.5	35.2 ± 21.3	25.5–45.0	66.5 ± 47.3	29.1–72.1
***BP***										
Controls	89.2 ± 15.1	87.3–90.9	80.4 ± 16.2	78.3–82.5	80.4 ± 19.6	78.7–82.1	68.9 ± 21.3	66.3–71.6	72.0 ± 23.4	69.4–74.6
RA	27.8 ± 14.0	24.1–32.4	27.7 ± 16.3	23.6–31.8	28.9 ± 18.4	26.2–34.6	29.9 ± 15.4	27.2–32.6	30.5 ± 17.2	26.1–34.9
SA	43.0 ± 28.1	16.9–69.0	47.2 ± 15.7	41.7–52.8	44.9 ± 16.5	41.6–48.1	49.8 ± 17.3	40.9–58.7	32.0 ± 18.3	27.9–36.0
Per PsA	43.1 ± 10.7	26.1–60.1	40.7 ± 17.3	28.3–53.1	35.8 ± 17.8	30.0–41.6	35.4 ± 20.2	28.4–42.5	34.3 ± 17.9	25.7–42.9
Ax PsA	53.7 ± 10.9	36.4–71.0	54.3 ± 27.2	33.3–75.2	46.2 ± 17.0	39.7–52.6	40.1 ± 10.4	35.3–44.8	40.0 ± 19.0	37.5–43.5
***GH***										
Controls	74.3 ± 15.1	72.5–76.1	62.7 ± 19.7	60.1–65.3	61.9 ± 16.5	60.5–63.4	53.6 ± 14.8	51.8–55.4	49.1 ± 15.5	47.4–50.8
RA	44.3 ± 20.9	39.2–49.2	42.5 ± 15.3	38.6–46.5	46.3 ± 20.4	43.3–49.3	41.4 ± 18.8	38.1–44.6	39.7 ± 20.5	34.5–45.0
SA	50.6 ± 32.2	20.8–80.3	52.5 ± 24.5	43.8–61.2	46.1 ± 19.9	42.1–50.0	50.1 ± 27.1	36.1–64.0	23.3 ± 18.0	14.3–42.3
Per PsA	60.0 ± 19.1	45.4–74.5	53.5 ± 12.2	44.7–62.2	48.4 ± 18.1	42.5–54.3	41.0 ± 13.0	36.4–45.5	35.7 ± 18.9	24.7–46.6
Ax PsA	68.7 ± 13.1	47.8–89.6	52.2 ± 13.4	41.8–62.5	40.3 ± 14.3	34.8–45.7	40.7 ± 16.8	33.0–48.3	40.0 ± 14.1	27.0–67.0
***MH***										
Control	71.6 ± 12.8	70.0–73.1	62.0 ± 14.3	60.1–63.8	64.2 ± 16.3	62.8–65.7	59.8 ± 17.2	57.7–61.9	58.7 ± 18.2	56.7–60.7
RA	44.1 ± 24.3	35.9–52.2	53.6 ± 21.4	48.1–59.0	53.2 ± 23.0	49.8–56.7	45.8 ± 22.0	42.0–49.6	51.6 ± 26.1	44.9–58.3
SA	48.0 ± 28.7	21.4–74.5	55.9 ± 22.4	47.9–63.8	54.5 ± 19.9	50.5–58.4	55.5 ± 20.0	45.1–65.8	46.6 ± 20.3	25.3–68.0
Per PsA	39.0 ± 22.9	12.4–75.5	44.4 ± 25.6	26.0–62.7	43.3 ± 16.8	37.9–48.8	45.4 ± 16.9	39.5–51.3	48.8 ± 17.9	38.4–59.2
Ax PsA	49.0 ± 18.0	20.3–77.6	59.6 ± 19.0	45.0–74.2	43.7 ± 20.8	35.7–51.6	47.6 ± 19.0	38.9–56.3	48.0 ± 45.2	28.5–54.5
***RE***										
Controls	85.3 ± 28.6	81.8–88.6	82.6 ± 30.1	78.6–86.5	80.7 ± 28.8	78.2–83.2	51.5 ± 41.5	46.4–56.6	54.8 ± 43.3	50.1–59.6
RA	47.7 ± 41.2	33.9–61.4	38.1 ± 42.1	27.4–48.8	39.5 ± 42.4	33.3–45.8	32.0 ± 38.5	25.3–38.7	41.2 ± 42.7	30.2–52.2
SA	46.5 ± 43.0	16.6–86.2	47.2 ± 28.6	37.0–57.3	40.2 ± 26.6	34.9–45.4	41.6 ± 27.9	27.2–55.9	38.7 ± 13.7	24.3–53.1
Per PsA	24.8 ± 16.5	20.3–51.1	46.6 ± 42.1	16.4–76.7	25.6 ± 26.8	16.9–34.3	24.4 ± 28.8	14.4–34.5	30.9 ± 30.5	13.2–48.5
Ax PsA	33.3 ± 47.1	21.6–58.3	48.0 ± 24.3	29.2–66.7	40.7 ± 27.3	30.3–51.1	28.5 ± 24.2	17.4–39.5	50.0 ± 24.0	36.0–66.0
***SF***										
Controls	78.2 ± 18.7	75.9–80.4	73.6 ± 19.6	71.1–76.2	72.7 ± 19.3	71.0–74.4	68.1 ± 19.9	65.6–70.6	66.0 ± 20.8	63.7–68.3
RA	38.1 ± 20.4	31.3–44.9	47.9 ± 19.1	43.1–52.8	50.7 ± 22.4	47.4–54.0	43.3 ± 20.1	39.8–46.8	47.5 ± 21.1	42.0–52.9
SA	51.4 ± 35.4	18.6–84.1	58.7 ± 22.6	50.7–66.8	54.0 ± 18.5	50.3–57.6	60.2 ± 21.7	49.1–71.4	33.2 ± 17.0	15.3–51.1
Per PsA	53.2 ± 29.3	16.6–79.8	44.9 ± 10.6	37.3–52.4	39.7 ± 20.9	32.9–46.5	44.1 ± 20.9	36.8–51.4	35.5 ± 19.3	32.1–43.8
Ax PsA	56.1 ± 21.7	21.4–90.8	51.2 ± 22.1	34.2–68.2	42.5 ± 7.1	39.8–45.3	42.7 ± 27.5	39.3–46.2	33.5 ± 19.1	30.0–42.0
***VT***										
Controls	62.4 ± 13.4	60.8–64.0	54.2 ± 15.5	52.2–56.3	57.7 ± 14.8	56.4–59.0	55.1 ± 15.2	53.3–57.0	53.6 ± 16.6	51.8–55.5
RA	31.6 ± 18.7	25.3–37.8	46.2 ± 19.5	41.2–51.1	44.6 ± 20.9	41.5–47.7	38.9 ± 20.9	35.3–42.6	42.2 ± 19.7	37.2–47.3
SA	45.0 ± 23.9	22.8–67.1	52.4 ± 20.4	45.1–59.6	47.8 ± 17.2	44.4–51.2	49.7 ± 20.4	39.2–60.2	39.1 ± 21.7	16.3–62.0
Per PsA	50.0 ± 18.1	35.4–64.5	55.0 ± 14.3	44.7–65.2	44.4 ± 16.4	39.1–49.8	41.7 ± 15.9	36.1–47.3	46.7 ± 13.8	38.8–54.7
Ax PsA	63.7 ± 33.5	10.4–87.0	49.4 ± 15.7	37.3–61.5	39.8 ± 15.5	33.9–45.7	36.4 ± 18.2	28.1–44.7	47.5 ± 38.8	31.9–66.9
***SF-36 PCS***										
Controls	54.9 ± 5.6	53.2–55.6	52.7 ± 6.1	51.9–53.5	50.8 ± 8.1	50.1–51.5	46.8 ± 8.2	45.8–47.8	43.8 ± 8.1	42.9–44.7
RA	32.3 ± 4.4	29.8–34.8	31.3 ± 5.4	29.9–32.7	31.5 ± 6.3	32.5–34.4	32.9 ± 6.0	30.8–34.9	31.0 ± 5.3	29.6–32.3
SA	37.6 ± 10.3	27.9–47.1	38.7 ± 8.5	35.7–41.7	37.0 ± 7.4	35.6–38.5	37.9 ± 12.3	31.6–44.2	24.8 ± 5.9	18.6–31.0
Per PsA	46.0 ± 3.3	40.7–51.2	37.7 ± 7.0	32.7–42.7	33.4 ± 6.7	31.2–35.6	33.2 ± 6.9	30.8–35.7	32.0 ± 3.5	30.0–34.0
Ax PsA	47.2 ± 3.2	42.0–52.4	40.3 ± 7.8	34.3–46.4	36.4 ± 6.6	33.9–38.9	35.4 ± 5.4	32.9–37.9	42.0 ± 13.4	28.4–62.4
***SF-36 MCS***										
Controls	47.7 ± 6.9	46.9–48.5	46.6 ± 6.7	45.7–47.5	46.3 ± 7.2	45.7–46.9	43.0 ± 9.4	41.8–44.1	43.6 ± 9.5	42.5–44.6
RA	40.6 ± 9.9	37.3–43.9	40.1 ± 11.4	37.2–44.0	40.7 ± 12.3	38.8–42.5	38.7 ± 10.7	36.8–40.6	38.6 ± 13.1	36.2–41.0
SA	39.0 ± 13.8	26.2–51.8	42.1 ± 11.1	38.1–46.0	40.2 ± 8.4	38.5–41.9	41.6 ± 10.9	35.9–47.2	38.9 ± 8.7	29.8–48.1
Per PsA	32.9 ± 4.5	25.7–40.0	40.6 ± 9.6	33.7–47.5	36.3 ± 6.1	34.3–38.3	36.0 ± 6.3	33.8–38.2	38.9 ± 7.3	34.7–43.2
Ax PsA	37.0 ± 6.7	26.3–47.7	41.0 ± 9.5	33.7–48.4	35.7 ± 7.4	32.9–38.6	35.3 ± 6.9	32.2–38.5	36.3 ± 12.6	27.0–42.7

Abbreviations: RA = Rheumatoid arthritis, SA = Ankylosing spondylitis, Per PsA = Peripheal Psoriatic arthritis, Ax PsA = Axial Psoriatic arthritis

**Table 4:** SF-36 subscales and summary scores in patients and the controls with primary school, secondary school and high school/university. Data are expressed as means ± SD and 95% CI

	***Eucational level***								
	**Primary school**			**Secondary school**			**High school/university**		
	*Mean*	*SD*	*95% CI*	*Mean*	*SD*	*95% CI*	*Mean*	*SD*	*95% CI*

***PF***									
Controls	75.4	21.8	73.8 – 77.1	89.3	14.1	88.1 – 90.5	91.4	13.0	89.4 – 93.3
RA	39.2	20.5	35.7 – 42.7	40.4	20.5	37.9 – 42.9	50.0	19.1	45.8 – 54.3
SA	51.0	25.5	43.1 – 58.9	52.6	17.0	47.4 – 57.8	55.9	18.9	45.8 – 66.0
Per PsA	42.3	24.9	33.0 – 51.6	42.0	18.9	37.3 – 46.7	55.0	19.3	25.0 – 75.0
Ax PsA	44.6	17.4	34.9 – 54.3	52.3	19.3	46.1 – 58.5	56.1	12.9	46.1 – 66.0
***RP***									
Control	65.5	38.3	62.6 – 68.4	80.8	32.1	78.0 – 83.5	83.8	30.3	79.4 – 88.3
RA	28.5	13.6	27.2 – 31.9	29.1	15.9	26.1 – 31.1	35.3	18.9	31.0 – 39.5
SA	33.1	33.2	28.9 – 49.3	39.6	25.9	30.7 – 46.5	54.6	31.9	37.6 – 71.6
Per PsA	23.6	25.4	14.1 – 33.0	37.3	25.3	31.0 – 43.5	68.7	37.5	19.0 – 88.4
Ax PsA	33.3	30.8	16.2 – 50.4	36.2	23.7	28.6 – 43.8	58.3	27.9	36.8 – 79.8
***BP***									
Controls	72.3	21.6	70.6 – 73.9	84.2	17.5	82.7 – 85.8	86.1	15.9	83.8 – 88.5
RA	29.3	17.8	27.3 – 33.3	30.3	16.4	27.3 – 32.4	31.6	17.3	27.7 – 35.5
SA	42.6	18.1	38.0 – 49.2	43.9	12.8	39.0 – 46.8	53.5	19.6	43.0 – 63.9
Per PsA	39.5	20.3	31.9 – 47.1	33.4	16.2	29.4 – 37.4	53.1	18.1	24.1 – 82.0
Ax PsA	35.1	14.5	27.0 – 43.2	46.4	13.5	42.1 – 50.8	64.1	18.8	49.6 – 78.6
***GH***									
Controls	55.2	18.2	53.8 – 56.6	64.3	17.9	62.7 – 65.8	68.9	14.8	66.7 – 71.1
RA	40.9	19.1	37.7 – 44.2	44.3	19.5	41.8 – 46.7	48.1	20.2	43.6 – 52.6
SA	45.2	22.5	38.3 – 52.2	45.4	20.8	39.0 – 51.7	57.2	21.3	45.8 – 68.6
Per PsA	44.6	16.0	38.6 – 50.6	45.5	17.8	41.1 – 49.9	48.7	2.5	44.7 – 52.7
Ax PsA	38.6	15.2	30.1 – 47.1	44.8	17.9	39.1 – 50.6	48.3	9.6	40.8 – 55.7
***MH***									
Controls	59.8	16.9	58.5 – 61.1	66.7	15.5	65.3 – 68.0	66.6	15.4	64.3 – 68.9
RA	48.8	24.2	44.7 – 52.9	49.8	23.1	46.9 – 52.6	54.2	22.0	49.3 – 59.1
SA	42.9	19.4	36.9 – 48.9	52.9	16.9	47.7 – 58.0	66.70	13.7	59.4 – 74.0
Per PsA	46.4	13.8	41.2 – 51.5	43.6	19.7	38.7 – 48.5	44.0	11.3	25.9 – 62.0
Ax PsA	39.2	22.2	26.8 – 51.5	48.7	20.1	42.2 – 55.1	54.7	17.1	41.6 – 67.9
***RE***									
Controls	63.2	40.5	60.1 – 66.3	79.3	33.2	76.4 – 82.2	79.4	31.0	74.9 – 84.0
RA	35.7	42.5	31.4 – 45.9	38.8	40.0	33.8 – 43.8	44.1	43.3	34.5 – 53.8
SA	33.3	26.5	25.1 – 41.4	40.1	26.0	32.2 – 48.0	77.1	31.5	60.2 – 93.9
Per PsA	21.0	25.4	11.5 – 30.5	31.2	27.0	22.5 – 32.9	28.9	31.9	25.8 – 35.6
Ax PsA	31.0	26.6	16.2 – 45.8	41.2	28.8	31.9 – 50.4	53.2	23.6	41.0 – 61.3
***SF***									
Controls	68.4	19.8	66.9 – 69.9	74.6	20.2	72.9 – 76.3	77.4	18.2	74.7 – 80.1
RA	44.7	21.0	41.1 – 48.3	47.2	21.6	44.5 – 49.9	48.8	20.2	44.3 – 53.3
SA	46.9	19.0	41.1 – 52.8	52.2	19.5	46.2 – 58.1	75.6	19.7	65.1 – 86.1
Per PsA	44.6	19.5	37.2 – 51.9	41.5	18.6	36.9 – 46.1	62.5	17.6	34.3 – 90.6
Ax PsA	39.0	8.0	34.6 – 43.5	45.8	10.8	42.3 – 49.3	49.8	18.8	35.4 – 64.3
***VT***									
Controls	55.1	15.4	53.9 – 56.2	58.8	15.7	57.4 – 60.1	57.7	14.1	55.6 – 59.8
RA	39.4	20.6	35.9 – 42.9	41.9	20.9	39.3 – 44.5	45.8	20.1	41.4 – 50.3
SA	40.9	20.8	34.5 – 47.3	48.2	15.9	43.4 – 53.1	54.6	11.1	48.7 – 60.6
Per PsA	47.6	15.1	41.9 – 53.3	43.6	16.4	39.5 – 47.6	48.7	10.3	32.3 – 65.1
Ax PsA	29.3	19.0	18.7 – 39.8	45.0	18.6	39.0 – 50.9	47.2	15.2	35.5 – 58.9
***SF-36 PCS***									
Controls	47.2	8.7	46.5 – 47.9	52.2	7.2	51.6 – 52.8	53.6	5.4	52.8 – 54.4
RA	31.7	5.9	30.7 – 32.7	32.2	5.8	31.4 – 32.9	34.5	6.2	33.1 – 35.8
SA	36.1	9.9	34.1 – 40.2	38.4	6.5	35.4 – 41.4	38.3	9.8	33.0 – 43.5
Per PsA	33.4	8.1	30.4 – 36.4	33.6	5.2	32.3 – 34.9	48.4	5.0	40.3 – 56.5
Ax PsA	35.0	6.0	31.6 – 38.3	37.5	7.1	35.2 – 39.7	43.0	4.2	39.8 – 46.2
***SF-36 MCS***									
Controls	44.1	8.3	43.4 – 44.7	47.1	7.9	46.4 – 47.7	46.5	7.4	45.4 – 47.6
RA	38.8	12.1	36.7 – 40.8	39.2	11.5	37.7 – 40.6	40.8	12.0	38.1 – 43.5
SA	34.8	7.4	32.5 – 37.1	39.9	7.9	37.5 – 42.3	50.4	8.8	45.7 – 55.1
Per PsA	37.1	4.7	35.3 – 38.8	37.0	7.6	35.1 – 38.9	31.7	6.6	21.1 – 42.4
Ax PsA	32.5	8.7	27.6 – 37.3	37.6	7.6	35.1 – 40.0	40.9	7.3	35.3 – 44.6

For definitions see Table 3

### Factors associated with poor health-related quality of life

Multiple regression models were constructed to adjust for factors potentially associated with poor HRQL in the four IRD groups. Covariates chosen *a priori *included the demographic variables, disease related characteristics and the and average score of the SCQ questionnaire. All these factors were introduced as covariates in multiple regression models in which PCS and MCS SF-36 summary scores (instead of a single subscale) were dependent variables. The physical component of the SF-36 was influenced by a high disease activity (measured by DAS) and chronic comorbidity (both at a p level < 0.0001), and by radiographic damage (p = 0.004) in RA. A similar association of chronic comorbidity and high disease activity with AS, peripheral PsA, and axial PsA were also found. Concerning the mental component, an association was found in RA with the disease activity (p < 0.0001), and in AS and axial PsA with the low educational level (p level at < 0.001 and 0.009, respectively). The severity of psoriatic lesions (assessed using PASI) was significantly associated with poor mental functioning in patients with peripheral and axial PsA (p level at < 0.0001 and 0.03, respectively).

## Discussion

Patient-reported outcomes (PRO) are an attractive option in a busy medical practice, as the time burden is transferred from the clinician to the patient [34]. The validity and usefulness of PRO data in evaluating and monitoring patients with IRD have been well documented [35,36]. PRO includes physical function or disability, pain, general health status, side effects, medical costs and other content areas. Inherent in the strategy of intensive treatment with Disease Modyifing Anti-Rheumatic Drugs – DMARDs (including biological agents) with the goal of preventing or slowing permanent structural joint damage and long-term disability in IRD is the accurate monitoring of HRQL in daily practice and in clinical trials [37]. The SF-36 is, to date, the most used tool that evaluates HRQOL as a subjective perception about psychological and physical limitations due to musculoskeletal disorders [38,39]. The summary scales PCS and MCS were chosen to represent HRQL in this study because they have been shown to be among the most valid SF-36 scales for measuring physical and mental health, respectively [40]. These scales are easier to administer and less expensive than physician-observed disease activity and process measures [35,36].

The results of this study show that adults with IRD have poorer self-reported health status than those without arthritis in all domains of living, but particularly with respect to scales measuring aspects of physical functioning or mobility, role limitation due to physical health problems and usual activities, and bodily pain. The disease with the worst HRQL for physical dimensions of SF-36 was RA. The mean PCS score for RA patients was 32.5, approximately two standard deviations below the mean observed in the Italian general population [25,38]. Based on the PCS scores alone, the physical functioning of these patients is comparable to patients with congestive heart failure [40-42]. This results was similar in men and women. Concerning patients with PsA and AS, our data confirms clinical cohort studies from Germany [22], United Kingdom [43], Turkey [44], and Canada [13], that found similar functional disability and reduced HRQL in patients with PsA compared to RA. Although patients with PsA reportedly had lower levels of physical disability by the SF-36 PCS, in comparison with health controls, they also reported more psychosocial problems than patients with RA and AS. Overall, the SF-36 MCS dimensions typically affected by PsA were mental health, limitations due to emotional health, and social functioning. The extent of disability among these patients may be attributed to the fact that these patients have an inflammatory skin condition as well as peripheral and/or axial joint disease [11,13]. The psychological and social effects of skin involvement have been well documented in patients with psoriasis [45,46]. In a survey by the National Psoriasis Foundation almost 75% of patients believed that psoriasis had moderate to large negative impact on their quality of life, with alterations in their daily activities [47]. Furthermore, physical and emotional affects of psoriasis were found to have a significant negative impact at patients' workplace. Fortune et al. [48] identified that pathological worry and anxiety occur in at least a third of patients with psoriasis and that psychological interpersonal difficulties impinge on all aspects of the patient's daily life. Other studies reported that between 5 and 20 percent of psoriasis patients had contemplated suicide [49,50]. When compared with patients with other diseases, such as cancer, arthritis, hypertension, heart disease, diabetes, and depression, patients with psoriasis reported a similar reduction in HRQL [42,51]. According to Chorus, et al. [45], we found that the physical component scores were more favourable in AS than in RA. However, there was a sex related difference: women reported lower scores than men in role limitations due to physical function and in general health subscales. These results were consistent with a previous study of Dagfinrud, et al. [52].

The findings of the multivariate analysis suggests that the SF-36 PCS scores may reflect both functional limitations due to current disease activity due to processes that do not respond to aggressive treatment with anti-rheumatic drugs and limitations due to the radiographic damage and coexisting conditions. Kirwin [53], similarly, concluded that disease activity remains the major determinant of disability in RA, both late in disease and in patients with substantial radiographic damage. Similarly, in psoriatic patients, the results of Husted, et al. [12] support the view that the disease activity was a significant predictor of physical functioning, as measured by the Health Assessment Questionnaire (HAQ) over the course of PsA, although its effect diminished over time.

The outcomes of a chronic condition may be also affected by coexisting chronic conditions. It is important to incorporate assessment of comorbidity into studies involving HRQL outcomes for persons with multiple chronic medical conditions, as coexisting conditions may substantially affect outcomes of interest such as physical functioning, overall health status, depression and response rates in randomized controlled trials [53-55]. Our patients accurately reported a majority of common comorbid conditions respect to the general population. In particular, 53.7% of our RA patients reported at least one comorbid condition, which is in accord with the studies of Rupp, et al. (56%) [56]. Berkanovic, et al. (54%) [57] and Gabriel, et al. (49.3%) [58]. Similarly, AS and PsA were associated with a variety of extra-articular manifestations that can result in a number of comorbid conditions. Comparison of the prevalence of comorbidity in these conditions remains, therefore, difficult, however, because the definition of comorbidity and the number of comorbid conditions included varied between the studies, and different comorbidity measures were used in all studies. Many comorbidity instruments were developed for hospitalized patients to adjust for mortality rates. These instruments may have limitations in adjusting for functional status as an outcome. Measures of comorbidity typically use information from the medical record or administrative data. These approaches impose limitations, such as the availability of medical records and the quality of documentation. Research has shown that patients can accurately assess their current and past medical conditions including comorbidities [59,60]. The SCQ, that added items about treatment (as a surrogate for disease severity) and functional limitation. represents an efficient method to assess comorbid conditions in clinical and health services research [32].

Of the demographic factors studied, education level had the most important association with negative impact on patients' mental HRQL among patients with chronic pain-associated disability. Despite its recognized importance in health outcomes, education level and other measures of socioeconomic status have been infrequently examined as predictors of quality of life in IRD. Lower levels of formal education have been reported to be a risk factor for presence of chronic musculoskeletal pain and physical function in the community and has been associated with a higher prevalence of work disability and greater disease activity in patients with AS and RA [9,38]. The mechanism by which education influences pain disability or psychological process is unclear but may be related to enhanced self-efficacy and sense of control allowing the patient to take advantage of a greater number of pain reducing modalities. Our findings suggest that educational level may have a greater effect on mental health outcomes in AS and axial PsA.

This study has several limitations that should be taken into account in interpreting the results. First, it is based in a tertiary referral Centre and patients with more severe IRD may be overrepresented. These results may, therefore, not be generalizable to all patients with IRD in the community. In addition, recall periods for the various measures differed. This discrepancy in recall time is common when using multiple self-report measures and is inherent in the measures. However self-report data are a valuable resource, and the problems encountered with self-report data are similar to those encountered in other forms of data collection. Second, the cross-sectional design limits the analysis about the associated factors with physical function and HRQL and does not allow to draw final conclusions about the strengths of the cause-effect relationships. The most of the literature on this issue are cross-sectional studies and not suited for statement or implications. Further, selection bias cannot be excluded. However, the relatively large group of patients was aged between 20 and 82 years, and the whole range of disease activity, physical functioning, and radiographic damage scores was represented, indicating a representative group of IRD patients.

## Conclusion

Despite to the limitations discussed above, our study confirms that the physical aspects of health seem to be most severely affected in patients with IRD although all dimensions of health were significantly affected, and in the PsA group of patients, the disease impact on mental health was considerable. A management programme for patients with IRD and the planning of the healthcare services should take these findings into account by maintaining the focus on impairment and physical function, but also focusing on the mental and social consequences of the disease. Longitudinal studies are, also, needed to examine how these quality of life measures change over time and respond to clinical and public health interventions.

## Abbreviations

AS: Ankylosing Spondylitis; BASDAI: Bath Ankylosing Spondylitis Disease Activity index; BP: Bodily Pain; CI: Confidence Interval; DAS: Disease Activity Score; DMARDs: Disease Modyifing Anti-Rheumatic Drugs; GH: General Health; HRQOL: Health-Related Quality of Life; IRD: Inflammatory Rheumatic Diseases; MAPPING: MArche Pain Prevalence INvestigation Group; MCS: Mental Component Summary; MH: Mental Health; mSASSS: Modified Stoke Ankylosing Spondylitis Spine Score; PASI: Psoriasis Area and Severity Index; PCS: Physical Component Summary; PF: Physical Functioning; PRO: Patient-Reported Outcomes; PsA: Psoriatic Arthritis (PsA); RA: Rheumatoid Arthritis; RE: Role limitations due to emotional health; RP: Role limitations due to physical function; SCQ: Self-Administered Comorbidity Questionnaire; SD: Standard Deviation; SF: Social Functioning; SF-36: Short Form 36-item Health Survey; s-Score: Standardized difference scores; VT: Vitality.

## Competing interests

The authors would like to make the following statements with regard to their conflicts of interest/financial disclosures: MI was a full-time employee of Bristol-Myers Squibb's Italy, at the time of study completion. WG is a consultant for Bristol-Myers Squibb, Abbott Immunology, General Electric, Esaote and Shering-Plough, has received honorarium from Bristol-Myers Squibb, Abbott Immunology, General Electric, Schering-Plough and Wyeth, and has received research support from Abbott Immunology and Wyeth. The rest of the authors have any financial or other competing interests.

## Authors' contributions

FS was the primary researcher, was responsible for co-ordinating and managing the study on a day-to-day basis, for data collection, data analysis and input into writing the manuscript. MC provided radiological support for the study, was involved in designing the study and helped draft the manuscript. SG provided clinical support and was involved in designing the study. MI contributed to revising the manuscript. WG helped in the design and the interpretation and was involved in critically revising the important intellectual content of the document. All authors have read and approved the final manuscript.
